# The Effect of Dentin Contamination by Topical Anesthetics on Micro-Shear Bond Strength: An In Vitro Experiment

**DOI:** 10.3390/ijerph192416567

**Published:** 2022-12-09

**Authors:** Nattawit Niyomsujarit, Pathomrat Uttamang, Meghna Burad, Nadaprapai Sipiyaruk, Kawin Sipiyaruk

**Affiliations:** 1 Department of Operative Dentistry and Endodontics, Faculty of Dentistry, Mahidol University, Bangkok 10400, Thailand; 2 Phibun Mangsahan Hospital, Ubon Ratchathani 34110, Thailand; 3 Mahidol International Dental School, Faculty of Dentistry, Mahidol University, Bangkok 10400, Thailand; 4 Private Practice, Bangkok 10400, Thailand; 5 Department of Orthodontics, Faculty of Dentistry, Mahidol University, Bangkok 10400, Thailand

**Keywords:** dentin bonding, dental education, dental practice, pain management, patient-centered care, shear bond strength, topical anesthetics

## Abstract

Topical anesthetics are commonly used to minimize pain and anxiety during dental procedures. Research is scarce on the influence of topical anesthetics on bond strength. Thus, this research evaluated the effect of dentin contamination by topical anesthetic solution and gel on the micro-shear bond strengths of etch-and-rinse and self-etch bonding systems. Ninety transversally hemi-sectioned dentin discs were prepared and randomly assigned to three groups: no contamination (control group), contamination with topical anesthetic solution (Xylonor spray, Septodont), and contamination with topical anesthetic gel (Xylonor gel, Septodont). Each contamination group was subdivided into two subgroups (*n* = 15) based on whether the adhesive system was etch-and-rinse (Optibond Solo Plus, Kerr) or self-etch (Optibond XTR, Kerr). Tygon tubes with resin composite (Filtek Z350 XT, 3M ESPE) were placed on each surface and light cured. After 24 h, a universal testing machine was used to measure micro-shear bond strength (MPa). Furthermore, nine additional specimens of non-contaminated and contaminated dentin were prepared and scanned by a scanning electron microscope. The data of micro-shear bond strength were analyzed using two-way ANOVA, and narrative analysis was used to qualitatively interpret visual data of the micro-morphology of dentin from the scanning electron microscope. No significant differences in micro-shear bond strength among different contamination groups and adhesive systems were found (*p* > 0.05). The results are supported by micro-morphology of the treated dentin surfaces and modes of failure, as the micro-morphology was similar among contamination and control groups. There was no significant impact of topical anesthetic forms and dentin bonding systems on the micro-shear bond strength, which was supported by the micro-morphology from a scanning electron microscope.

## 1. Introduction

Pain management appears to be the determination of the success of dental treatment due to its significance in the development of dental anxiety and behavior management. Topical anesthesia is frequently employed to alleviate factors that lead to uneasiness in a patient through its pharmacological effects [1], which can relieve the anxiety of patients following their pain reduction. Similar to local anesthesia, the mechanism of topical anesthesia is based on the inhibition of nerve conduction as the influx of sodium ions at the sodium channel is blocked at the site of administration. Nerve transmission recovers from the inhibition after the distribution and excretion of the anesthetic [2]. After a topical anesthetic is applied for a duration of 2 min [3], the anesthesia extends 2–3 mm into the mucosa in terms of depth [4] and lasts for 45–60 min [5]. The efficacy of a topical anesthetic substantially depends on numerous factors, including forms of topical anesthetic, which is commonly seen in solution, gel, ointment, or patch form.

The restoration of a tooth surface using resin composite requires mechanical adhesion, a result of infiltration by dental adhesives and precipitated through resin tags and collagen fibers in dentin, and chemical adhesion, caused by the interaction between the acidic monomer and hydroxyapatite in dentin. Dental adhesives are divided into two systems, namely etch-and-rinse and self-etch system [6,7]. Acid dissolves the mineral content in dentin and enamel, while eliminating smear layer and smear plug by 5 to 8 μm in depth [8]. Collagen fibers and dentinal tubules open afterwards to allow the adhesive bonding to penetrate through microporous enamel. Polymerization assists mechanical adhesion as it leads to the formation of resin tags that infiltrate dentinal tubules and a hybrid layer comprising adhesive bonding, collagen fibers and hydroxyapatite [9]. However, moisture must be preserved in collagen fibers after etching to prevent collapse, which would lead to incomplete infiltration of adhesion and may result in tooth sensitivity.

According to the adhesion-decalcification concept, there appears to be an enamel and dentin progress interaction between the functional monomer and calcium in hydroxyapatite to form a calcium salt. The stability of the calcium salt generated influences the chemical adhesion and dissolution [10]. Previous research indicates that contamination of tooth surface with saliva and blood during adhesive application reduces adhesion between the tooth surface and restorative material [11,12,13,14] and significantly reduces bond strength [15]. Dentin contaminated with aluminum chloride hemostatic solution indicates no significant influence on shear strength with etch-and-rinse adhesives but demonstrates significantly lower shear strength when self-etch adhesives are applied [16]. Therefore, the contamination of topical anesthetics may block the penetration of bonding adhesives into the dentinal tubules to form resin tags, which could possibly affect shear bond strength.

Topical anesthetics can be used to relieve pain and discomfort in gingival cord retraction or placement of a matrix band and wedge, which are common procedures in restorative dental practice. Pain relief during such procedures can be alleviating and supportive for dental patients with certain conditions. Patients of dental students commonly present with anxiety and fear of pain related to previous dental experiences [17]. In addition, as cord retraction requires experience and practice, the goal of ensuring a pain-free experience for the patients may be stressful for dental students [18]. As gingival retraction packing during tooth restoration can be painful depending on the sensitivity of patients and skills of dental professionals, the application of topical anesthetics for this procedure should be considered, especially among inexperienced dentists and dental students.

Since the effects of contamination of topical anesthetics on bonding of etch-and-rinse and self-etch adhesives are yet to be thoroughly explored, this study aimed to investigate the effect of the dentin contamination from two different forms of topical anesthetics (solution and gel) on the micro-shear bond strength of dentin for etch-and-rinse and self-etch adhesive systems. Therefore, a null hypothesis of this research was set as “the dentin contamination with topical anesthetics would not significantly affect the bond strength of a resin-based material”. This research also qualitatively evaluated whether or not there were any differences in the micro-morphology of the differently treated dentin surfaces.

## 2. Materials and Methods

### 2.1. Study 1: Micro-Shear Bond Strengths through a Universal Testing Machine in Different Dentin Preparations

#### 2.1.1. Tooth Specimen Preparation

Forty-five human premolars were extracted for orthodontic treatment within three months prior to this research and it was ensured that these teeth had no cavity, restoration, crack, or fracture. Using the low speed cutting machine (Isomet, Buehler, KA, USA) with water spray, the teeth were immersed in 0.1% Thymol solution and were horizontally sliced, perpendicular to the long axis of the tooth. The tooth was initially cut at the crown four millimeters above the cementoenamel junction, followed by a second slice which was performed horizontally two millimeters below the first slice. The surface of the tooth was polished through water using a 600-grit silicon carbide grinding paper (Buehler^TM^, Lake Bluff, IL, USA) for a duration of one minute to control the amount of smear layer. Subsequently, the slide was cut in half in the bucco-lingual plane, producing forty-five pairs of slides. These slides were then separated into two groups based on whether the etch-and-rinse (OptiBond^TM^ Solo^TM^, Kerr Corp., Orange, CA, USA) or the self-etch adhesive systems (OptiBond^TM^ XTR, Kerr Corp., Orange, CA, USA) was employed for each one. The slides were then further divided into three sub-groups with the first group being contaminated with a topical anesthetic in its solution form (Xylonor Spray, Septodont, Cedex, Saint-Maur-des-Fossés, France), the second group being contaminated with a topical anesthetic in its gel form (Xylonor Gel, Septodont, Cedex, Saint-Maur-des-Fossés, France), and a control group (Table 1). Bonding was then applied on these slides.

#### 2.1.2. Tooth Surface Preparation

##### ERC Group: Etch-and-Rinse Adhesive Applied to Non-Contaminated Dentin (Control Group)

The tooth surface was rinsed thoroughly with water for 30 s to eliminate any contaminant. Excessive water was absorbed for 10 s. The tooth surface was etched with 37.5% Phosphoric acid for 15 s and rinsed thoroughly with water for 15 s. Excessive water was absorbed for 10 s for suitable moisture. Etch-and-rinse bonding was applied using a micro brush in a rubbing motion for 15 s, following the manufacturer’s instruction. The tooth surface was blown for 5 s with the syringe tip placed at one centimeter.

##### ERS Group: Etch-and-Rinse Adhesive Applied to Dentin Contaminated with Topical Anesthetic Solution

The tooth surface was applied with a topical anesthetic solution for 2 min and rinsed with water for 30 s. Excessive water was absorbed for 10 s. The tooth surface was etched with 37.5% Phosphoric acid for 15 s and rinsed thoroughly with water for 15 s. Excessive water was absorbed for 10 s for suitable moisture. Etch-and-rinse bonding was applied using a micro brush in a rubbing motion for 15 s, following the manufacturer’s instruction. The tooth surface was blown for 5 s with the syringe tip placed at one centimeter.

##### ERG Group: Etch-and-Rinse Adhesive Applied to Dentin Contaminated with Topical Anesthetic Gel

The tooth surface was applied with a topical anesthetic gel for 2 min and rinsed with water for 30 s. Excessive water was absorbed for 10 s. The tooth surface was etched with 37.5% Phosphoric acid for 15 s and rinsed thoroughly with water for 15 s. Excessive water was absorbed for 10 s for suitable moisture. Etch-and-rinse bonding was applied using a micro brush in a rubbing motion for 15 s, following the manufacturer instruction. The tooth surface was blown for 5 s with the syringe tip placed at one centimeter.

##### SEC Group: Self-Etch Adhesive Applied to Non-Contaminated Dentin (Control Group)

The tooth surface was rinsed thoroughly with water for 30 s to eliminate any contaminant. Excessive water was absorbed for 10 s. The primer was applied using a micro brush in a rubbing motion for 20 s. The self-etch adhesive was applied following the manufacturer’s instruction. The tooth surface was blown for 5 s with the syringe tip placed at one centimeter.

##### SES Group: Self-Etch Adhesive Applied to Dentin Contaminated with Topical Anesthetic Solution

The tooth surface was applied with a topical anesthetic solution for 2 min and rinsed with water for 30 s. Excessive water was absorbed for 10 s. The primer was applied using a micro brush in a rubbing motion for 20 s. The self-etch adhesive was applied following the manufacturer’s instruction. The tooth surface was blown for 5 s with the syringe tip placed at one centimeter.

##### SEG Group: Self-Etch Adhesive Applied to Dentin Contaminated with Topical Anesthetic Gel

The tooth surface was applied with a topical anesthetic gel for 2 min and rinsed with water for 30 s. Excessive water was absorbed for 10 s. The primer was applied using a micro brush in a rubbing motion for 20 s. The self-etch adhesive was applied following the manufacturer’s instruction. The tooth surface was blown for 5 s with the syringe tip placed at one centimeter.

#### 2.1.3. Micro-Shear Bond Strength Test

Each plastic Tygon tube (size 0.8 mm in diameter, 0.8 mm in width and 1.0 mm in height) filled with a resin composite (Filtek Z350 XT, 3M ESPE) of the shade A2 was placed on the prepared tooth surface next to the dentin-enamel junction and then light cured for 20 s. It was then soaked in water at the temperature of 37 degrees Celsius for 24 h, and sliced for inspection for defects, such as voids, using a 100× polarizing microscope. The defective specimens were then excluded.

To assess the micro-shear bond strength [19], each specimen was fixed in a universal testing machine using cyanoacrylate glue. A wire sized 0.2 mm in diameter was placed at the junction between the resin composite and the dentin. Micro-shear bond strength (MPa) was measured with a cross head speed of one millimeter/minute until the specimen dislodged.

### 2.2. Study 2: Dentin Micro-Morphology through a Scanning Electron Microscope in Different Surface Preparations

Nine additional specimens were prepared to study dentin using a 3500× microscope when placed in different preparations as below:

(1) Non-contaminated dentin
(2) Non-contaminated dentin with 37.5 percent phosphoric acid applied on
(3) Non-contaminated dentin with self-etching primer applied on
(4) Dentin contaminated with topical anesthetic solution
(5) Dentin contaminated with topical anesthetic solution and 37.5 percent phosphoric acid applied on
(6) Dentin contaminated with topical anesthetic solution and self-etching primer applied on
(7) Dentin contaminated with topical anesthetic gel
(8) Dentin contaminated with topical anesthetic gel and 37.5 percent phosphoric acid applied on
(9) Dentin contaminated with topical anesthetic gel and self-etching primer applied on

These nine specimens were soaked in a fixative solution and then rinsed with 2.5 percent Glutaraldehyde in 0.1 M Sorensen phosphate buffer (pH 7, 4 degrees Celsius) for 12 h. These specimens were then rinsed with 0.2 M Sorensen phosphate buffer for an hour, followed by distilled water for one minute. Evaporation was then performed by soaking the specimens in ethanol with a concentration of 25, 50, 75, 95 and 100 percent for 20, 20, 20, 10 and 50 min, respectively. Subsequently, the specimens were soaked in Hexamethyldisilazane (HDMS) for 10 min and then dried for 10 min. The slides were then covered with gold to be scanned by a scanning electron microscope.

### 2.3. Statistical Analysis

Two-way ANOVA was performed to investigate the influence of topical anesthetics and dentin bonding systems on micro-shear bond strength. Statistical significance was taken at *p* < 0.05. The micro-morphology of dentin from a scanning electron microscope was evaluated using narrative analysis, which is an analytic method used to qualitatively interpret visual data.

### 2.4. Ethical Consideration

This research was granted exemption by the Faculty of Dentistry/Faculty of Pharmacy, Mahidol University, Institutional Review Board, reference number MU-DT/PY-IRB 2017/016.1705.

## 3. Results

### 3.1. Micro-Shear Bond Strengths in Different Dentin Preparations

**Figure 1:**
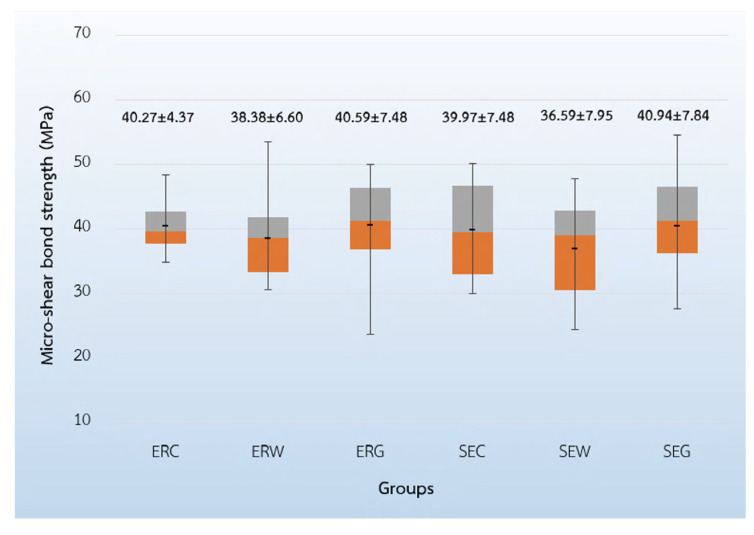
Micro-shear bond strength of the six specimens (MPa).

The findings demonstrated that the self-etch adhesive-applied dentin contaminated with topical anesthetic gel (SEG) group yielded the highest micro-shear bond strength at 40.94 ± 7.84 MPa. The second and the third highest for micro-shear bond strength were the etch-and-rinse adhesive-applied dentin contaminated with topical anesthetic gel (ERG) group at 40.59 ± 7.48 MPa, and the control group, which was the etch-and-rinse adhesive-applied to non-contaminated dentin (ERC) group, at 40.27 ± 4.37 MPa, respectively. The self-etch adhesive-applied dentin contaminated with topical anesthetic solution (SES) group provided the lowest micro-shear bond strength at 36.59 ± 7.95 MPa. These findings are presented in Figure 1. Two-way ANOVA indicated that the contamination of topical anesthetics in different forms and different adhesive systems had no influence on micro-shear bond strength (*p* > 0.05), as demonstrated in Table 2.

### 3.2. Micro-Morphology of the Treated-Dentin Surfaces

**Figure 2:**
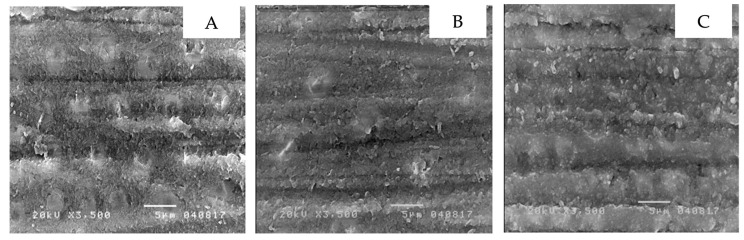
The dentin micro-morphology with a thick smear layer over the dentin surface: non-contaminated dentin (**A**), dentin contaminated with topical anesthetic solution (**B**), dentin contaminated with topical anesthetic gel (**C**).

**Figure 3:**
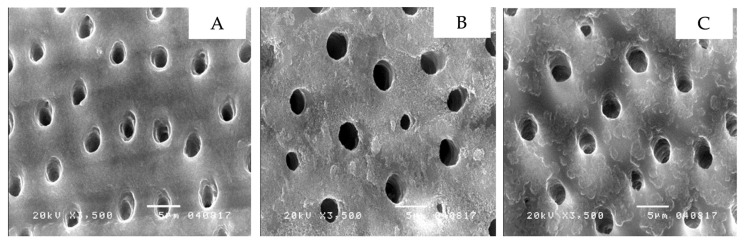
The dentin micro-morphology with 37.5 percent phosphoric acid applied to demonstrate an absence of a smear layer or smear plug, widely opened dentinal tubules, and a presence of silica particles as shown in the image: non-contaminated dentin (**A**), dentin contaminated with topical anesthetic solution (**B**), dentin contaminated with topical anesthetic gel (**C**).

**Figure 4:**
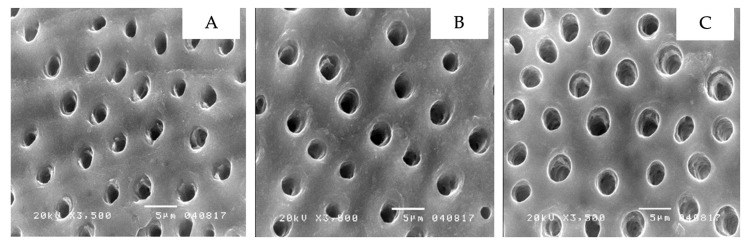
The dentin micro-morphology with self-etching primer applied to demonstrate a clean surface with widely open dentinal tubules: non-contaminated dentin (**A**), dentin contaminated with topical anesthetic solution (**B**), dentin contaminated with topical anesthetic gel (**C**).

The scanning electron microscope showed the micro-morphology of the differently treated-dentin surfaces at 3500× magnification. Non-contaminated dentin, grinded with 600-grit sandpaper, had a thick smear layer over the dentin surface that led to the dentinal tubules not being visible (Figure 2A). Additionally, dentin contaminated with topical anesthetic solution (Figure 2B), or topical anesthetic gel (Figure 2C) demonstrated comparable results. In contrast, non-contaminated dentin with 37.5 percent phosphoric acid applied displayed the absence of a smear layer or smear plug, widely open dentinal tubules and the presence of silica particles, a component of phosphoric acid gel (Figure 3A). This was further found in dentin contaminated with topical anesthetic solution (Figure 3B), and topical anesthetic gel (Figure 3C), which was applied with 37.5 percent phosphoric acid. Furthermore, non-contaminated dentin applied with the self-etching primer displayed a clean surface and widely open dentinal tubules (Figure 4A). A similar result was seen in dentin contaminated with topical anesthetic solution (Figure 4B), and topical anesthetic gel (Figure 4C), which had the self-etching primer applied to it.

## 4. Discussion

This study found that contamination of topical anesthetics did not influence the micro-shear bond strength. With the exception of eucalyptus oil and spearmint oil, all ingredients in both topical anesthetic solution and gel are hydrophilic, thus easily dissolved and rinsed by water. Since a small proportion of both eucalyptus oil and spearmint oil are incorporated for purposes of flavoring, despite being hydrophobic this does not interrupt bonding and is readily eliminated by phosphoric acid or the self-etching primer. Similarly, previous research stated that lubricating oil employed for dental handpiece maintenance demonstrated no effect on the micro-shear bond strength of dental adhesives in both etch-and-rinse (Single Bond^®^) and self-etch systems (Clearfil SE Bond^®^ and One-Up Bond F^®^), as acid in the etch-and-rinse system could eliminate and dissolve the oil on dentin while acid in the self-etch system could reduce the effect of oil contamination [20].

The use of the etch-and-rinse adhesive on dentin contaminated with either topical anesthetic solution or gel did not yield significantly different micro-shear bond strengths. Correspondingly, Aluminum chloride, commonly used as a hemostatic agent, had no influence on the micro-shear bond strength of etch-and-rinse adhesives (Excite^®^) [16]. This is due to the pH of phosphoric acid being 0.5 or lower, which allows it to clear dentin of contamination as it can dissolve minerals in both enamel and dentin and eliminate smear layer and smear plug as deep as 5–8 μm [16]. Furthermore, this study demonstrated that the use of self-etch adhesive on dentin contaminated with either a topical anesthetic solution or gel did not yield significantly different micro-shear bond strengths. Comparably, previous literature reported no significant difference between the bond strength of non-contaminated dentin and dentin contaminated with saliva, both before and after the use of self-etch adhesives, due to the bifunctional acid monomer in self-etch being hydrophilic, which aids bonding with dentin and provides lower sensitivity to contamination [21]. The composite’s shear bond strength when dentin is contaminated with silane and has self-etch adhesive applied to it does not degrade [22]. In contrast, aluminum chloride significantly reduced the micro-shear bond strength of the self-etch adhesives [16]. However, silane contamination demonstrated an adverse effect on the dentin bond strength of etch-and-rinse adhesives [23]. In addition, the application of a self-etching primer for 40 s in comparison to 20 s significantly increased micro-shear bond strength [16]. Therefore, the type of contamination agent and adhesive system are influential factors that affect micro-shear bond strength.

There appeared to be similarities in the micro-morphology of the differently treated dentin surfaces for both the control and the contamination groups. To begin with, a thick smear layer over dentin was found despite being rinsed in water [24]. In the control group, the smear layer and smear plug were eliminated after the application of phosphoric acid and were accompanied by open dentinal tubules and silica particles. As for the contamination groups, slightly different outcomes were identified as silica particles were absent, but open dentinal tubules and the absence of the smear layer and smear plug was identified. The absence of silica particles may be due to topical anesthetic gel or solution remaining partially present on the dentin surface after being rinsed. Lidocaine is a secondary amide that possesses negative ions which leads to the bonding of oxygen and silicon, a positive ion, allowing it to be washed away [25]. For dentin surfaces with self-etching primer applied, comparable results were visible in non-contaminated dentin and in dentin contaminated with anesthetic gel and solution, as open dentinal tubules were present and the smear layer/plug was absent. This concludes that the contamination of topical anesthetic gel or solution does not influence dentin surfaces with self-etching primer or phosphoric acid applied.

The contamination of dentin by topical anesthetic gel and solution prior to the application of either etch-and-rinse or self-etch adhesives does not affect the micro-shear bond strength, which leads to the successful application of topical anesthetics for pain reduction in various procedures, inclusive of rubber dam and retraction cord application [26]. Therefore, the application of topical anesthetics should be considered for these procedures, especially among inexperienced dentists and dental students to assure a pain-free experience for their patients. This will be very supportive for patients who may have fear and anxiety toward dental treatments. However, the dentin specimens in this research were prepared using silicon carbide grinding that generates a loose smear layer, compared to the ones prepared using a bur-cutting technique. This loose smear layer can be easily dissolved by the moderate acidic self-etching adhesive used in this research with a pH of 1.6. Thus, the micro-morphology from the scanning electron microscope revealed a clean surface with widely open dentinal tubules prepared by self-etching primer and proved to be similar to the one prepared by phosphoric acid, which was much more acidic. In addition, we did not simulate oral conditions when preparing the specimens. Consequently, further research of long-term adhesions in vitro such as cyclic loading or thermocycling should be required. Moreover, an additional form of anesthetics or adhesive systems should be further investigated.

## 5. Conclusions

This in-vitro research revealed no significant impact of topical anesthetic forms and dentin bonding systems on the micro-shear bond strength of dentin. In addition, there appeared to be similarities in the micro-morphology of the differently treated dentin surfaces for both the contamination and control groups. Consequently, this research supports the use of topical anesthetics for cord retraction packing during tooth restoration, as there is no negative effect on the shear bond strength of both adhesive systems. However, further experiments in simulated oral environments should be required to confirm the impact of topical anesthetics on dentin shear bond strength.

## Figures and Tables

**Table 1:** Sample groups (*n* = 15 specimens for each group).

Sample Group	Etch-and-Rinse System	Self-Etch System
Non-contaminated dentin (control group)	ERC	SEC
Dentin contaminated with topical anesthetic solution	ERS	SES
Dentin contaminated with topical anesthetic gel	ERG	SEG

**Table 2:** Two-way ANOVA for micro-shear bond strength (MPa) among the six groups of specimens.

Scheme	Sum of Squares	df	Mean Squares	F	*p*-Value
Bonding	7.64	1	7.64	0.153	0.697
Contamination	181.52	2	90.76	1.820	0.168
Interaction (Bonding * Contamination)	17.95	2	8.98	0.180	0.836
Error	4189.67	84	49.88		

## Data Availability

The data that support the findings of this study are available from the corresponding author, upon reasonable request.
